# Raman spectral band imaging for the diagnostics and classification of canine and feline cutaneous tumors

**DOI:** 10.1080/01652176.2025.2486771

**Published:** 2025-04-09

**Authors:** Mindaugas Tamošiūnas, Martynas Maciulevičius, Romans Maļiks, Diāna Dupļevska, Daira Viškere, Ilze Matīse-van Houtana, Roberts Kadiķis, Blaž Cugmas, Renaldas Raišutis

**Affiliations:** a Institute of Atomic Physics and Spectroscopy, University of Latvia, Rīga, Latvia; b Ultrasound Research Institute, Kaunas University of Technology, Kaunas, Lithuania; c Institute of Electronics and Computer Science, Riga, Latvia; d Department of Electrical Power Systems, Faculty of Electrical and Electronics Engineering, Kaunas University of Technology, Kaunas, Lithuania

**Keywords:** Raman spectral band imaging, near-infrared autofluorescence, veterinary oncology, mast cell tumor, soft tissue sarcoma

## Abstract

This study introduces Raman imaging technique for diagnosing skin cancer in veterinary oncology patients (dogs and cats). Initially, Raman spectral bands (with specificity to certain molecular structures and functional groups) were identified in formalin-fixed samples of mast cell tumors and soft tissue sarcomas, obtained through routine veterinary biopsy submissions. Then, a custom-built Raman macro-imaging system featuring an intensified CCD camera (iXon Ultra 888, Andor, UK), tunable narrow-band Semrock (USA) optical filter compartment was used to map the spectral features at 1437 cm^−1^ and 1655 cm^−1^ in *ex vivo* tissue. This approach enabled wide-field (cm^2^), rapid (within seconds), and safe (< 400 mW/cm^2^) imaging conditions, supporting accurate diagnosis of tissue state. The findings indicate that machine learning classifiers – particularly support vector machine (SVM) and decision tree (DT) – effectively distinguished between soft tissue sarcoma, mastocytoma and benign tissues using Raman spectral band imaging data. Additionally, combining Raman macro-imaging with residual near-infrared (NIR) autofluorescence as a bimodal imaging technique enhanced diagnostic performance, reaching 85 – 95% in accuracy, sensitivity, specificity, and precision – even with a single spectral band (1437 cm^−1^ or 1655 cm^−1^). In conclusion, the proposed bi-modal imaging is a pioneering method for veterinary oncology science, offering to improve the diagnostic accuracy of malignant tumors.

## 1. Introduction

Pet animals frequently develop skin and subcutaneous tumors. Mast cell tumors (MCTs) are among the most common malignant skin tumors in dogs. They have a prevalence rate of approximately 20% and originate from the immune mast cells (London and Thamm 2013). Mast cell tumors also develop in cats, however, they are less frequently malignant. Soft tissue sarcomas (STSs), which originate from connective tissue (i.e. mesenchymal origin) are other frequently occurring skin malignancies, i.e. 1–10 of every 10,000 vaccinated cats develop injection-site STS (Zabielska-Koczywąs et al. 2017).

Because 20–40% (or 70–80%) of diagnosed skin tumors in dogs (or cats) are malignant (Withrow et al. 2013) veterinarians need prompt and clinically feasible diagnostic methods. Despite advances in diagnostic macro imaging, including X-rays, computed tomography (CT), magnetic resonance imaging (MRI) and ultrasound (US), these methods have lower spatial resolution in comparison to optical imaging. Also, the MRI and CT equipment and procedures are costly, and these devices may not be accessible locally. Recently, optical coherence tomography (OCT) was validated for veterinary skin cancer diagnosis and intraoperative tumor imaging, offering <10 μm axial resolution, imaging depth up to 2 mm, and lateral resolution close to optical resolution. OCT enabled detecting the surgical margins *ex-vivo* of feline sarcoma (Coleman et al. 2021), canine mammary tumors (Fabelo et al. 2021), canine adenocarcinoma (Lapsley et al. 2022), canine mast cell tumors (MCT) (Dornbusch et al. 2021) and canine soft tissue sarcomas (STS) (Lages and Selmic 2021; Selmic and Ruple 2020). Pioneering OCT study by Mesa et al. designed effective machine learning (ML) algorithms for feline and canine sarcoma differentiation from muscle and adipose tissue by using a sliding window (SWA) feature extraction from OCT images (Mesa et al. 2017). As a next step, a collaborative study by Ye et al. (2021) introduced AI methods to identify cancerous tissue at surgical margins of canine STS, relying on CNN ResNet-50 feature extraction capability. With only a small amount of training data (80 cancer images, 80 normal images), and the validation dataset (20 cancer images, 20 normal images), a deep learning-based method was able to produce excellent sensitivity (98%) and specificity (97%) for intraoperative assessment of STS surgical margins. However, the application of OCT for clinical decision support also requires priced equipment starting at €70 k. Additionally, OCT has certain limitations; from our experience, OCT scattering artifacts manifest in almost all OCT B-scan images, measured in skin neoplasms (Cugmas et al. 2021). When these artifacts are filtered out during OCT image mask generation, the tissue area available for SWA is reduced, potentially leaving some tumor cell-containing locations unanalyzed by OCT SWA.

The implementation of machine learning (ML) opened the possibility for automated feature extraction and classification of the diagnostic medical macro images. In human medicine, ML has successfully diagnosed various types of cancer using bright-field dermatoscopy (Esteva et al. 2017), OCT combined with photoacoustic remote sensing (Ecclestone et al. 2021), autofluorescence lifetime imaging (Unger et al. 2020), ultrasound imaging in combination with multispectral imaging (Tiwari et al. 2020), and photoacoustic raster scanned imaging (Wong et al. 2017; Kang et al. 2022). In veterinary medicine, ML applications are still in early development. Only a few studies have used artificial intelligence for veterinary cancer image analysis for studying animal health in general (Ezanno et al. 2021; Li et al. 2020; Zuraw and Aeffner 2022; Celniak et al. 2023; Dank et al. 2023; Banzato et al. 2023; Burti et al. 2022; Dupļevska et al. 2024). Related our group study showed that linear discriminant analysis (LDA) with leave-one-out cross-validation diagnosed skin malignant tumors from *ex vivo* biopsy submissions with the sensitivity and specificity of 94% and 98%, using a multimodal dataset comprising OCT B-scan image parameters obtained from SWA and Raman spectral parameters (Tamošiūnas et al. 2022). However, diagnosing accurately between MCT and STS still remained a significant challenge.

The present research employed ML linear classifiers (LDA, SVM, NB, DT) earlier tested by R. Raišutis group for diagnosing human skin tumors in multispectral and ultrasonic imaging setups (Tiwari et al. 2020). The diagnostic method and technology developed during our study implementation is based on Raman and NIR-autofluorescence spectral band imaging at 1437 cm^−1^ and 1655 cm^−1^ narrow-band intervals. The experiments were carried out *ex vivo* on the neoplasms of veterinary patients (dogs and cats). The study aimed to determine whether alterations in spectral and intensity parameters of Raman scattering and NIR-autofluorescence images can be successfully applied to provide the diagnosis and classification of skin cancer.

## 2. Materials and methods

### 2.1. Tissue samples

The samples originated from client-owned dogs and cats for which the veterinarian-clinician recommended excisional surgery. Immediately after the surgery, tissue samples were immersed in 10% formalin solution and delivered to a veterinary pathology laboratory (Matise Veterinary Pathology service, Riga, Latvia). In the laboratory, gross characteristics of tissue size, shape, density and color were assessed and representative tissue sections were trimmed according to the published guidelines (Kamstock et al. 2011). Trimmed sections were routinely processed through a series of alcohols, embedded in paraffin, then 4 μm histological sections were cut, deparaffinized, stained with hematoxylin and eosin. A board-certified veterinary pathologist provided assessment of tissue histopathology, including a detailed assessment of the tumors (tumor cells, stroma, margins, malignancy and grade) to determine the definitive diagnosis. Tissue samples diagnosed as soft tissue sarcoma (12 dogs and 2 cats), mast cell tumor (8 dogs and 6 cats) along with adjacent non-cancerous tissue (from the same samples, where available) were provided to Biophotonics laboratory (University of Latvia), stored in 10% formalin. Thus, *ex vivo* samples from 14 mast cell tumors (MCTs), 14 soft tissue sarcomas (STSs), as well as 14 benign tissue samples were used for Raman spectroscopy and spectral band imaging (Appendix A).

### 2.2. Spectroscopy measurements

**Figure 1.**
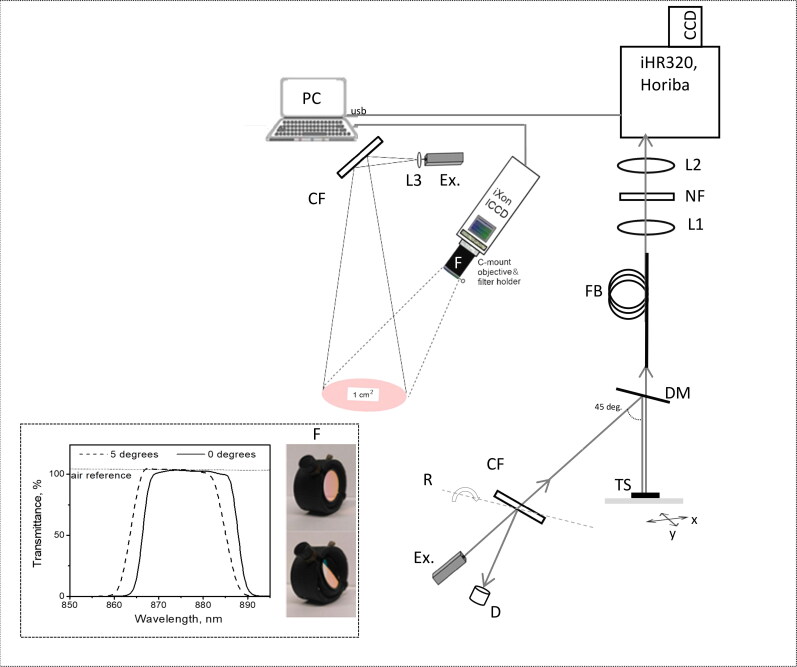
Combined set-up for the acquisition of Raman macro images and spectra. Intensified CCD camera (iXon Ultra 888, Andor) records the images through a set of optical filters, embedded into the filter holder (F), including 785 nm laser rejection filter (OD_785 nm_ >6), and two band pass filters rotated at calibrated angle pivot optical axis, which is mounted on 35 mm focal length, f/2.8–f/16 aperture objective. Additional components: Ex. – 785 nm laser; CF – laser clean-up filter; D – beam dump container; R – optomechanics to rotate CF filter pivot optical axis; DM – dichroic mirror; TS – tissue sample; xy – translational stage; FB – Raman signal collecting fiber; L1, L2 – fiber coupler lenses; L3 – laser beam expansion lens; NF – notch filter (OD785 nm >6). Insert: removable part of a filter holder (F) with filter FF01-880/11-25 at 0 degrees or at 5 degree pivot rotation angle. As indicated, angular tilt of tunable narrow-band filter by 5 degrees, produces ∼3 nm anti-Stokes shift at 90% of transmittance level. Transmittance measurements have been taken separately by DU800 spectrometer (Beckman Coulter, USA) at 0.2 nm wavelength accuracy.

As indicated in Figure 1, the system for single-point Raman spectra acquisition was built around iHR320 spectrograph equipped with 1200 g/mm grating and thermoelectrically cooled Syncerity-CCD camera (Horiba Jobin Yvon SAS, Longjumeau, France). A 785 nm laser excitation (Cobolt 08-NLD, Hübner, Kassel, Germany) illuminated the tissue surface area of ∼0.28 cm^2^ delivering the fluence rate of ∼388 mW/cm^2^. Laser clean-up filter (LD01-785/10-12.5, Semrock, USA) pivotal rotation was used to adjust the excitation light intensity (110 mW) delivered to the sample, with the rest laser power (390 mW) entering the beam dump container. The ultra-steep dichroic mirror (DM, #86-245, Edmund Optics, USA) served to reflect excitation light to the tissue sample (TS), same as to transmit the Raman signal to collecting fiber bundle (FB). The 785 nm excitation notch filter NF (NF03-785E-25, Semrock, USA) was additionally used to suppress the excitation signal. We collected Raman (+residual NIR autofluorescence) spectra for each tissue sample using 60 s scan time, 20 μm of spectrograph entrance slit aperture and full vertical binning of Syncerity-CCD camera, reaching a spectral resolution of 0.17 nm. The spectra were measured by placing *ex vivo* tissue sample (TS) under the laser beam, noting the sample orientation and the measurement site; afterwards, the sample was moved by using the micrometric positioning stage to deliver excitation light at a new random position, and the spectral measurements were repeated 3 times. For each spectrum, the residual NIR autofluorescence was separated from Raman signal using a 9^th^ to 16^th^ order polynomial fit, available in Labspec 6 software (Horiba, Jobin Yvon SAS, Longjumeau, France).

### 2.3. Imaging of selected spectral bands

We used a non-scanning, large area Raman spectral band imaging method, based on tunable band-pass filter application. Previously, such diagnostic method has been introduced by prof. W. Grundfest group in 2015 (University of California, USA), enabling to detect the microscopic bone like structures within the soft tissue (Papour et al. 2015). The differentiation between muscle and bone has been achieved due to the intense Raman band presence at 956 cm^−1^ ascribed to symmetric stretching of tetrahedral P–O bonds in phosphate (PO_4_)^3-^ group (Papour et al. 2015). We built a similar macro imaging system (Figure 1) comprising intensified CCD camera (iXon Ultra 888, Andor Technology, UK), a long pass emission filter (LP, #69-905, Edmund Optics, USA) and two interchangeable sets of Semrock (USA) narrow-band optical filters. These filters were embedded in a custom-made filter compartment (F), mounted in front of a macro lens (35 mm focal length, f/2.8–f/16, Edmund Optics, USA) allowing for the imaging of two separate spectral bands, centered at 1437 cm^−1^ or 1656 cm^−1^ (Tamošiūnas et al. 2023). As indicated in Figure 1 (insert), rotation of the tunable narrow-band Semrock filter (FF01-880/11-25) pivot optical axis shifts its transmittance to the anti-Stokes region. The filter transmittance measurements had been taken separately by using DU800 spectrometer (Beckman Coulter, USA) at 0.2 nm wavelength accuracy. Therefore, the concept of the imaging method is to capture two images: one at the Raman peak ­wavenumber (with NIR autofluorescence) and another at a shifted wavenumber without Raman scattering, which measures just the NIR autofluorescence. By subtracting the autofluorescence image from the Raman peak image, the background autofluorescence is removed, and the image of Raman scattered light is obtained.

During Raman spectral band imaging, each tissue sample was positioned with the same side and orientation as it was during spectral acquisition. The EMCCD camera was cooled to −30 °C; the slowest readout rate (50 kHz at 16-bit) was selected to generate less noise, and the acquisition mode was set to ‘single’ with optimized 10 s exposure time per image, at 300 gain. The excitation laser light reflected from CF filter (and thus not used in spectroscopy set-up) illuminated the tissue sample over the area of approximately 1 cm^2^, delivering a fluency rate of about 390 mW/cm^2^. During the imaging process, two spectral band images centered around 1437 cm^−1^ were captured:

1^st^ image – by calibrated rotation (pivot optical axis) of FF01-900/11-25 (Semrock, USA) filter positioned in a removable filter compartment beyond unrotated filter FF01-880/11-25. Such position of filters is abbreviated as ‘0-R’;

2^nd^ image – by calibrated angular rotation of both FF01-900/11-25 and FF01-880/11-25 filters. Such position of filters is abbreviated as ‘R-R’.

For Raman spectral band imaging centered around 1655 cm^−1^, the removable filter compartment with the FF01-880/11-25 filter was replaced with the FF01-910/5-25 filter compartment. Then, two spectral band images were captured again:

1^st^ image – by calibrated rotation of FF01-910/5-25 filter, positioned in front of unrotated FF01-900/11-25 filter; such position of filters is abbreviated as ‘R-0’.

2^nd^ image – by using both FF01-910/5-25 and FF01-900/11-25 filters in their unrotated positions; such position of filters is abbreviated as ‘0-0’.

By following this procedure, four Raman images acquired for each spectral band were respectively labelled as: ‘R-0’, ‘R-R’, ‘0-R’ and ‘0-0’. These labels will be further used in the Results section of the paper.

The white light images of the tissues were separately acquired using EMCCD camera with filter compartment (F) removed. This step enabled spatial mapping and co-localization of white light (WL) and Raman images obtained from the tissue.

### 2.4. Calibration of the narrowband filters rotation

**Figure 2.**
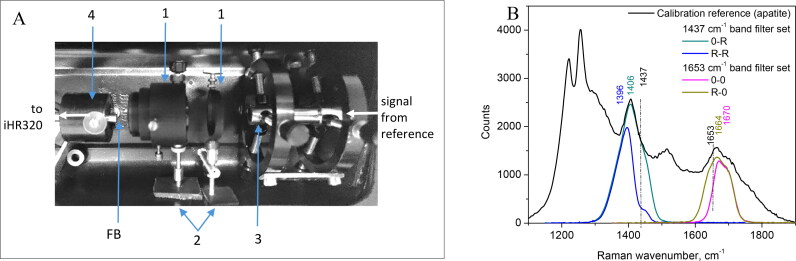
The cage set-up for narrowband filters ‘rotation-stop’ calibration (a) with two Raman band-pass filters mounted (1), filter rotation handles attached (2), fiber Y-bundle tip fixed for optical signal input (3) and signal collecting fiber (FB) mounted in the holder (4) which is aligned to the optical axis of the Y-bundle tip. Results of the filter transmittance calibration (b) used for separation of 1437 cm^−1^ or 1655 cm^−1^ Raman spectral band peaks from NIR autofluorescence signals.

We assembled a light-sealed calibration chamber, enabling to measure and mechanically fixate the narrowband filter rotational ‘stop’ position, by using the feedback from a spectroscopic measurement. The chamber set-up is depicted in Figure 2(a): it contains two mounts for Raman filter compartments (a), external handles for Raman bandpass filters rotation (b), Y-shaped, low-OH, fiber bundle tip (c) produced by Lightguide (Latvia) and aligned with the optical axis of Raman signal collecting fiber (FB) mounted in the holder (d). A geologic mineral apatite (Ca_5_(Cl,F)·(PO_4_)_3_) was used as the reference sample, exhibiting intense spectral peaks at 1406 cm^−1^ and 1663 cm^−1^ (Figure 2(b)). Filter calibration principle: a 785 nm laser light (Cobolt 08-NLD) was coupled to one leg of Y-shaped fiber (∅ 5.85 mm, 487 fibers, 200 μm core/fiber) for excitation of apatite reference. The optical signal from apatite was collected by ∅10 mm diameter (NA = 0.22) joint end of Y-shaped bundle placed in close vicinity to the reference sample. The reference optical signal was transmitted to calibration chamber by the second leg of the fiber bundle (∅ 8.2 mm, NA = 0.22), passed the filter compartment and entered the iHR320 spectrograph *via* collecting fiber (FB). The angular rotation of FF01-900/11-25 and FF01-880/11-25 filters, mounted in pair in a calibration chamber, shifted the narrowband spectral signal from 1437 cm^−1^ transmittance to anti-Stokes spectral region matching spectral band position from NIR autofluorescence alone at 1396 cm^−1^ (Figure 2(b)). The anti-Stokes shift was also obtained for FF01-910/5-25 and FF01-900/11-25 filters pair, shifting the narrowband spectral signal transmittance from 1670 cm^−1^ to 1664 cm^−1^, and as a result – increasing the transmittance at 1655 cm^−1^.

The final results of filters rotational ‘stop’ calibration are summarized according to iHR320 spectrograph readings considering 50% of signal transmittance for each spectral band. The set of FF01-900/11-25 and FF01-880/11-25 filters at ‘0-R’ position allowed capturing Raman images within 1365–1455 cm^−1^, or within 1358–1418 cm^−1^ wavenumber interval when used at ‘R-R’ position. The set of FF01-910/5-25 and FF01-900/11-25 filters at ‘R-0’ position allowed capturing Raman images within 1625–1715 cm^−1^; or within 1651–1716 cm^−1^ wavenumber interval when used at ‘0-0’ position. After the calibration procedure, the pivot rotational ‘stop’ position of each filter were permanently fixed.

Workflow for separating Raman spectral band images from NIR autofluorescence is given in Appendix B.

### 2.5. Feature extraction, training of the models, testing and evaluation

For subsequent feature extraction from Raman or autofluorescence spectral band images, 20 patches were registered from each type of image by applying rotation and translation operations. Each patch, representing a 1 cm^2^ square area, was saved in *.tiff format.

To characterize the texture features of the image patches, a grayscale co-occurrence matrix was generated. From this matrix, three quantitative parameters were calculated: entropy, energy (energy gr.) and homogeneity. The rest parameters: average, energy, standard deviation (st. dev.), root mean square (RMS), contrast, variance, skewness, and kurtosis were computed from the histogram of pixel intensities within image patches. All feature quantification was conducted using Matlab (Mathworks, USA) software.

The study utilized four supervised machine learning classifiers: Linear Discriminant Analysis (LDA), Support Vector Machine (SVM), Naive Bayes (NB) and Decision Tree (DT), as previously described in (Tiwari et al. 2020). To assess the general performance of the training and identify potential under-fitting, the models were tested on resubstituted samples. Following this, a standard 10-fold cross-validation was performed to assess possible over-fitting and simulate the models’ predictive performance on ‘unseen’ data. For the final testing of the model, the dataset was divided into separate training (90% of the data) and testing datasets (10%). In order to account for the tissue heterogeneity, we have performed 10 different training-testing splits (setting random number generator in Matlab to seed #1). The respective data, obtained from 10 confusion matrices, were cumulated into single. The summarized accuracy, sensitivity, specificity and precision metrics are presented in Tables 1–3.

**Table 1.** Performance metrics of different classification models for diagnosing between MCT and STS tumors.

#	Spectral band image data input				Classifiers				
	Raman		Autofluorescence		LDA	SVM	NB	DT	Diagnostic output
	1437 cm^–1^	1655 cm^–1^	1437 cm^–1^	1655 cm^–1^					
	MCT vs STS								
1	+				0.7679	0.7732	0.6393	0.8482	Accuracy
					0.8036	0.8143	1.0000	0.8571	Sensitivity
					0.7321	0.7321	0.2786	0.8393	Specificity
					0.7500	0.7525	0.5809	0.8421	Precision
2		+			0.7161	0.7696	0.5571	0.7929	Accuracy
					0.8571	0.8143	0.1607	0.8036	Sensitivity
					0.5750	0.7250	0.9536	0.7821	Specificity
					0.6685	0.7475	0.7759	0.7867	Precision
3	+	+			0.8071	0.8464	0.6268	0.8679	Accuracy
					0.8571	0.8571	0.9607	0.8607	Sensitivity
					0.7571	0.8357	0.2929	0.8750	Specificity
					0.7792	0.8392	0.5760	0.8732	Precision
4			+		0.8500	0.9000	0.6946	0.8375	Accuracy
					0.8214	0.9214	0.8429	0.8429	Sensitivity
					0.8786	0.8786	0.5464	0.8321	Specificity
					0.8712	0.8836	0.6501	0.8339	Precision
5				+	0.7804	0.8589	0.6714	0.8125	Accuracy
					0.7893	0.8500	0.6393	0.8179	Sensitivity
					0.7714	0.8679	0.7036	0.8071	Specificity
					0.7754	0.8655	0.6832	0.8092	Precision
6			+	+	0.8625	0.9571	0.6946	0.9054	Accuracy
					0.8786	0.9464	0.7964	0.9214	Sensitivity
					0.8464	0.9679	0.5929	0.8893	Specificity
					0.8512	0.9672	0.6617	0.8927	Precision
7	+		+		0.8732	0.9286	0.7232	0.8857	Accuracy
					0.8821	0.9321	0.9893	0.8857	Sensitivity
					0.8643	0.9250	0.4571	0.8857	Specificity
					0.8667	0.9255	0.6457	0.8857	Precision
8		+		+	0.7964	0.9554	0.6821	0.8946	Accuracy
					0.7893	0.9679	0.5214	0.9107	Sensitivity
					0.8036	0.9429	0.8429	0.8786	Specificity
					0.8007	0.9443	0.7684	0.8824	Precision
9	+	+	+	+	0.9179	0.9589	0.7125	0.9036	Accuracy
					0.9286	0.9571	0.9286	0.9179	Sensitivity
					0.9071	0.9607	0.4964	0.8893	Specificity
					0.9091	0.9606	0.6484	0.8924	Precision

The shaded values in lines 7 and 8 represent the outcomes of using only single spectral band imaging (1447 cm^-1^ or 1655 cm^-1^) to distinguish between MCT or STS cancer tissues and benign tissues.

**Table 2.** Performance metrics of different classification models for diagnosing between MCT and benign tissue.

#	Spectral band image data input				Classifiers				
	Raman		Autofluorescence		LDA	SVM	NB	DT	Diagnostic output
	1437 cm^–1^	1655 cm^–1^	1437 cm^–1^	1655 cm^–1^					
	MCT vs benign								
1	+				0.6982	0.8089	0.6571	0.7875	Accuracy
					0.8214	0.8571	0.9571	0.8071	Sensitivity
					0.5750	0.7607	0.3571	0.7679	Specificity
					0.6590	0.7818	0.5982	0.7766	Precision
2		+			0.6946	0.7286	0.5518	0.7929	Accuracy
					0.8214	0.7500	0.1786	0.7929	Sensitivity
					0.5679	0.7071	0.9250	0.7929	Specificity
					0.6553	0.7192	0.7042	0.7929	Precision
3	+	+			0.7821	0.8768	0.6804	0.8429	Accuracy
					0.8893	0.8821	0.8821	0.8607	Sensitivity
					0.6750	0.8714	0.4786	0.8250	Specificity
					0.7324	0.8728	0.6285	0.8310	Precision
4			+		0.6804	0.8804	0.6589	0.8143	Accuracy
					0.7464	0.8929	0.7964	0.8000	Sensitivity
					0.6143	0.8679	0.5214	0.8286	Specificity
					0.6593	0.8711	0.6246	0.8235	Precision
5				+	0.6446	0.7714	0.6393	0.7232	Accuracy
					0.6857	0.7750	0.6857	0.7107	Sensitivity
					0.6036	0.7679	0.5929	0.7357	Specificity
					0.6337	0.7695	0.6275	0.7289	Precision
6			+	+	0.7179	0.9589	0.6554	0.8536	Accuracy
					0.7679	0.9571	0.7071	0.8786	Sensitivity
					0.6679	0.9607	0.6036	0.8286	Specificity
					0.6981	0.9606	0.6408	0.8367	Precision
7	+		+		0.7750	0.9214	0.7107	0.8750	Accuracy
					0.8464	0.9107	0.9429	0.8750	Sensitivity
					0.7036	0.9321	0.4786	0.8750	Specificity
					0.7406	0.9307	0.6439	0.8750	Precision
8		+		+	0.7357	0.9179	0.5679	0.8750	Accuracy
					0.7643	0.9286	0.2929	0.8571	Sensitivity
					0.7071	0.9071	0.8429	0.8929	Specificity
					0.7230	0.9091	0.6508	0.8889	Precision
9	+	+	+	+	0.8232	0.9643	0.7071	0.8982	Accuracy
					0.8607	0.9643	0.8500	0.9036	Sensitivity
					0.7857	0.9643	0.5643	0.8929	Specificity
					0.8007	0.9643	0.6611	0.8940	Precision

The shaded values in lines 7 and 8 represent the outcomes of using only single spectral band imaging (1447 cm^-1^ or 1655 cm^-1^) to distinguish between MCT or STS cancer tissues and benign tissues.

**Table 3.** Performance metrics of different classification models for diagnosing between STS and benign tissue.

#	Spectral band image data input				Classifiers				
	Raman		Autofluorescence		LDA	SVM	NB	DT	Diagnostic output
	1437 cm^–1^	1655 cm^–1^	1437 cm^–1^	1655 cm^–1^					
	STS vs benign								
1	+				0.6964	0.7536	0.5696	0.8321	Accuracy
					0.6536	0.8036	0.2000	0.8250	Sensitivity
					0.7393	0.7036	0.9393	0.8393	Specificity
					0.7148	0.7305	0.7671	0.8370	Precision
2		+			0.5964	0.7161	0.4982	0.7268	Accuracy
					0.6679	0.7107	0.0643	0.7250	Sensitivity
					0.5250	0.7214	0.9321	0.7286	Specificity
					0.5844	0.7184	0.4865	0.7276	Precision
3	+	+			0.7411	0.8696	0.5500	0.8482	Accuracy
					0.7429	0.8821	0.2000	0.8464	Sensitivity
					0.7393	0.8571	0.9000	0.8500	Specificity
					0.7402	0.8606	0.6667	0.8495	Precision
4			+		0.7268	0.8804	0.6161	0.8732	Accuracy
					0.7607	0.8786	0.4536	0.8679	Sensitivity
					0.6929	0.8821	0.7786	0.8786	Specificity
					0.7124	0.8817	0.6720	0.8773	Precision
5				+	0.7411	0.8893	0.6304	0.7893	Accuracy
					0.7214	0.8857	0.8536	0.7893	Sensitivity
					0.7607	0.8929	0.4071	0.7893	Specificity
					0.7509	0.8921	0.5901	0.7893	Precision
6			+	+	0.8196	0.9589	0.6536	0.8821	Accuracy
					0.8036	0.9750	0.6929	0.8893	Sensitivity
					0.8357	0.9429	0.6143	0.8750	Specificity
					0.8303	0.9446	0.6424	0.8768	Precision
7	+		+		0.7893	0.9536	0.6589	0.8964	Accuracy
					0.7929	0.9500	0.4000	0.8750	Sensitivity
					0.7857	0.9571	0.9179	0.9179	Specificity
					0.7872	0.9568	0.8296	0.9142	Precision
8		+		+	0.7911	0.9554	0.5750	0.8625	Accuracy
					0.7536	0.9643	0.2929	0.8714	Sensitivity
					0.8286	0.9464	0.8571	0.8536	Specificity
					0.8147	0.9474	0.6721	0.8561	Precision
9	+	+	+	+	0.8750	0.9696	0.6357	0.9214	Accuracy
					0.8571	0.9786	0.4036	0.9214	Sensitivity
					0.8929	0.9607	0.8679	0.9214	Specificity
					0.8889	0.9614	0.7533	0.9214	Precision

The shaded values in lines 7 and 8 represent the outcomes of using only single spectral band imaging (1447 cm^-1^ or 1655 cm^-1^) to distinguish between MCT or STS cancer tissues and benign tissues.

### 2.6. Statistics

Statistical analysis included the unpaired nonparametric Mann–Whitney test to determine the significance between the calibration constants (k), implemented for Raman spectral band images, for the separation from NIR-autofluorescence at 1437 cm^−1^ (or at 1655 cm^−1^) between tissue groups (*n* = 14). One-way ANOVA test was used to compare the distribution of the ratio of NIR-autofluorescence spectral intensity at 1437 cm^−1^ (or at 1655 cm^−1^) to Raman spectral intensity values between tissue groups (*n* = 42). The averaged intensity distribution of Raman spectra was plotted as the mean (± standard error of the mean at the indicated spectral peaks). The spectral data from a single measurement is plotted as the mean ± SD.

## 3. Results

### 3.1. Separation between NIR autofluorescence and Raman spectral band images

**Figure 3.**
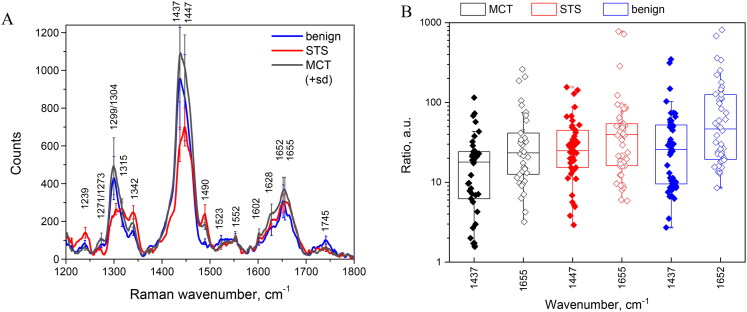
(a) Raman spectra values for skin, mast cell tumor (MCT), soft tissue sarcoma (STS) and benign tissue with annotated peaks. During the preprocessing of initial Raman spectral data, the residual autofluorescence curve was subtracted by applying the polynomial approximation and stored separately. For each spectrum (*n* = 42) the NIR-autofluorescence values at 1437 cm^−1^ (or 1447 cm^−1^) and 1652 cm^−1^ (or 1655 cm^−1^) were registered and divided with corresponding Raman spectral intensity values. The resulting distribution ratio of NIR-autofluorescence to Raman spectral intensity is depicted in (b). The mean values for ratio distributions at 1437 cm^−1^ (or 1655 cm^−1^) are not significantly different among MCT, STS, and benign tissues, as determined by one-way ANOVA.

Figure 3(a) shows Raman spectra of MCT, STS cancer tissues and adjacent benign tissue, with subtracted autofuorescence background. The mean spectra from benign and cancerous tissues are characterized by the most intense Raman band peaking at 1437–1447 cm^−1^ corresponding to CH_2_ and CH_3_ vibration modes in proteins and lipids (De Gelder et al. 2007; Talari et al. 2015). Raman spectra also show increased intensity at 1652–1655 cm^−1^ originating from C=O stretching in primary amides and C=C stretching in lipids, also C–O stretching model of amide in keratin. The bands around 1301, 1239 cm^−1^ and 1342 cm^−1^ can be attributed to C–H vibrations in lipids, proteins (collagen), also to C–N and N–H vibrations in tertiary amides. Other spectral intensity bands originate from guanine (1315 cm^−1^), tryptophan (1552 cm^−1^), phenylalanine (1602 cm^−1^), primary amides (1628 cm^−1^) and lipids (1745 cm^−1^). Raman peak at 1490 cm^−1^ originates from formalin previously observed in fixed benign and cancerous tissues (Talari et al. 2015).

Under biological tissue excitation at 785 nm, high intensity autofluorescence is superimposed with Raman photons. As evidenced by Figure 3(b), NIR-autofluorescence spectral signal is one or two orders of magnitude stronger in comparison to Raman emission intensity, with the broad distribution of intensity ratio values even for the same tissue class. Thus, the resulting imaging signal on the EMCCD detector can be also seen as the sum of Raman scattering and NIR-autofluorescence spectral intensities for the transmittance interval of narrowband filters used.

**Figure 4.**
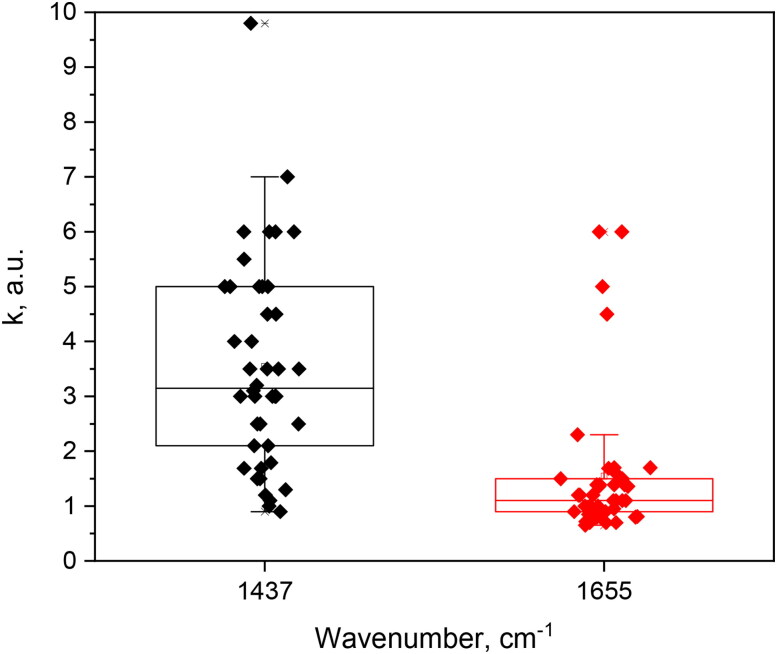
Distribution of multiplicator constants (k) used to extract the Raman spectral band images from NIR-autofluorescence. The meaning of k: the spectral integral values between ‘0-R’and ‘R-R’ spectral band images (for 1437 cm^−1^) or between ‘R-0’and ‘0-0’ (for 1655 cm^−1^ band) could not be equalized *via* ‘rotation-stop’ calibration (Figure 2(b)); therefore the multiplicator constant k is applied to ‘R-R’ or ‘0-0’ image (which has the lower spectral integral value) to increase its NIR-autofluorescence intensity (*k* > 0). Thus, before the final image of Raman scattered light is computed by removing the background NIR-autofluorescence, the calibration constant k is always applied on one of the images (‘R-R’ or ‘0-0’). Afterwards, the average intensity ratio between NIR-autofluorescence and Raman images is compared to corresponding spectral intensity ratio. In case there is no match between the ratios, the manual iteration process to apply the k value is started again, until the final Raman spectral band image is obtained, matching the spectral intensity ratio. The mean values for k distributions at 1437 cm^−1^ (or at 1655 cm^−1^) were not significantly different among MCT, STS, and benign tissues, according to Mann-Whitney test (*n* = 14).

The challenge of developing the Raman spectral band imaging technique starts with the separation of the Raman spectral signal from NIR-autofluorescence. As proposed by Papour et al. (2015) the final Raman image can be calculated by subtracting two images at different peak-transmitted wavenumbers, thereby separating the NIR-autofluorescence background and the Raman spectral band signals. Inspired by this study, earlier we tested a slightly modified workflow for separation of Raman spectral band images from NIR-autofluorescence, introducing the fixed calibration constants (k) for autofluorescence intensity compensation, both for 1437 cm^−1^ and 1655 cm^−1^ spectral band images (Tamošiūnas et al. 2023). In this study, Figure 4 illustrates the distribution of calibration constants (k) implemented for Raman spectral band image separation from NIR-autofluorescence, obtained from 14 samples from separate biopsy submissions. Data indicated the significant heterogeneity for autofluorescence intensity compensation constants (k) needed to be applied on ‘R-R’ (or ‘0-0’) images, even for the same tissue type (MCT, STS or benign), prior to autofluorescence subtraction. The results indicate that a tissue type specific and fixed multiplicator constant cannot be established for separating Raman spectral band images from NIR-autofluorescence. Therefore, autofluorescence compensation on ‘R-R’ or ‘0-0’ images requires to be performed iteratively and should be guided by Raman and NIR autofluorescence spectral intensity measurements taken at a time of spectral band imaging implementation. Although this was presumed, it was not directly evident before conducting our experiments. In our set-up, these 3 spectral measurements took additional 3 min per tissue sample *ex vivo*.

### 3.2. Imaging results

**Figure 5.**
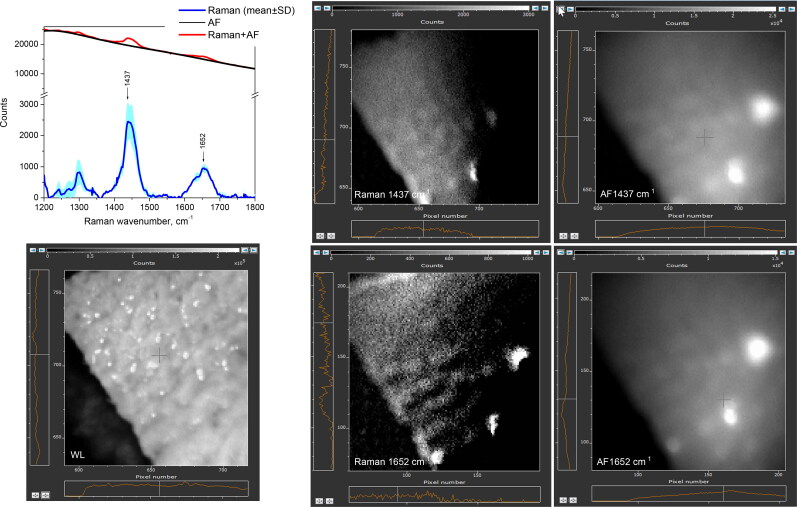
Skin tissue imaging results from sample #25(adjacent to STS tumor). Autofluorescence or Raman images, computed at 1437 cm^−1^ and 1652 cm^−1^ peak wavenumbers, are displayed. White light image field of view is 1.4 cm × 1.4 cm. To validate the imaging results, Raman spectral data (w/o fluorescence subtracted) are included.

Figure 5 displays a representative *ex vivo* images of the benign tissue (skin) after separating between NIR-autofluorescence spectral band and Raman spectral band images at 1437 cm^−1^ or 1652 cm^−1^ peak wavenumbers. The average intensity of AF-1437cm^−1^ computed image was 2 × 10^4^ a.u., in comparison to 1.2 × 10^2^ a.u. for AF-1652 cm^−1^ image, which is in agreement with the spectral data showing the autofluorescence intensity decay towards the longer wavenumbers. Raman-1437 cm^−1^ and 1652 cm^−1^ images show significantly reduced and more uniform signals, respectively at 0.3–2.8 (× 10^3^) and 0.1–1.3 (× 10^3^) a.u. pixel intensity values. The parts of the 1437 cm^−1^ and 1652 cm^−1^ images where Raman signal is brighter are likely caused by non-uniform laser beam intensity illumination. The obtained imaging result is in agreement with the spectral data; it also show that Raman band at 1652 cm^−1^ possesses weaker intensity, in comparison to the 1437 cm^−1^ band.

**Figure 6.**
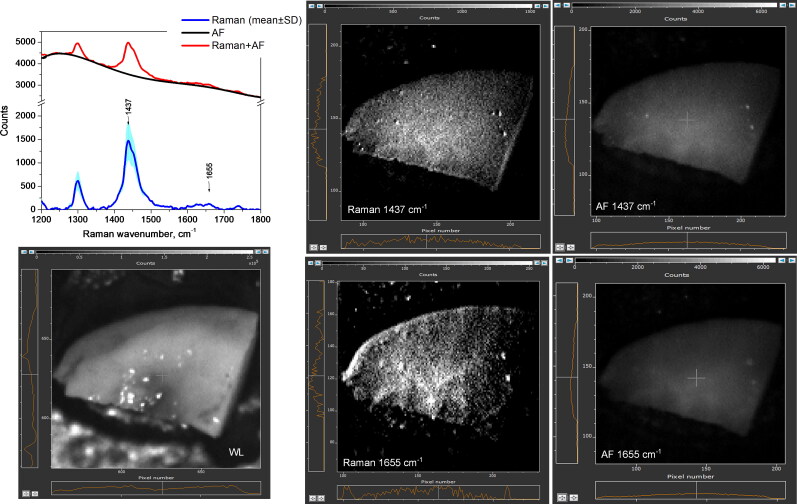
Mast cell tumor tissue imaging in sample #9 (sphynx cat, Appendix A). Computed Raman spectral band images and NIR autofluorescence (AF) images at 1437 cm^−1^ and 1655 cm^−1^ peak wavenumbers are displayed. White light (WL) image field of view is 2 cm × 2 cm. To validate the imaging results, Raman spectral data (w/o autofluorescence subtracted) are included.

According to Figure 6 and the examination of MCT images, the calculated Raman signals at 1437 cm^−1^ and 1655 cm^−1^ possess intensity range within 0.1–1.3 (× 10^3^) a.u., and 0.1– 0.4 (× 10^3^) a.u., respectively. The autofluorescence image pixel values, both at 1437 cm^−1^ and 1655 cm^−1^ wavenumbers reach the intensities up to 6 × 10^3^ a.u. The obtained MCT imaging results also agree with the spectral data, showing that Raman signal is weaker than NIR autofluorescence, and providing the proof that the intensity of both autofluorescence and Raman spectral bands images decay as the wavenumbers increase.

**Figure 7.**
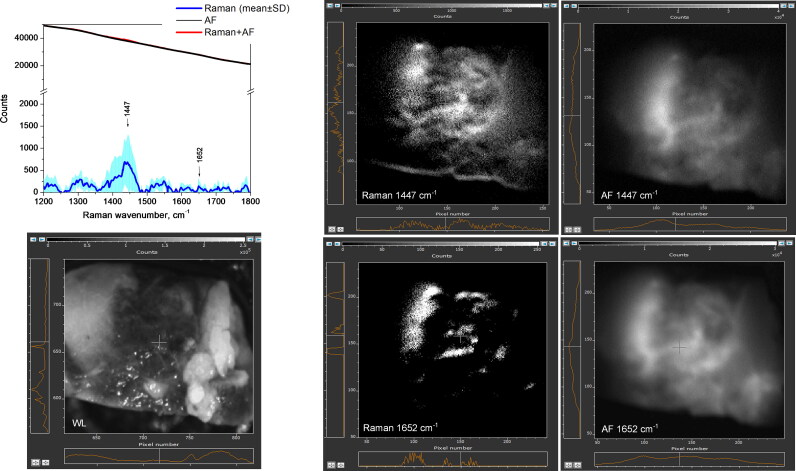
Soft tissue sarcoma tumor imaging (sample #16, mix breed cat, Appendix A). Computed Raman spectral band images and NIR autofluorescence (AF) images at 1447 cm^−1^ and 1652 cm^−1^ peak wavenumbers are displayed. White light (WL) image field of view is 1.5 cm × 1.5 cm. To validate the imaging results, Raman spectral data (w/o autofluorescence subtracted) are included.

In agreement to spectral findings (Figure 7), the accompanying imaging and analysis of STS samples, Raman signals at both 1447 cm^−1^ and 1652 cm^−1^ exhibited the lowest intensity levels. One of the artifacts, noticed in the computed Raman signal image, is the masking effect induced by the strong NIR-autofluorescence of STS (up to 2.4 × 10^4^ a.u.) on the weak STS Raman signal. Consequently, the lack of a discernible Raman signal occurred in that specific region, especially for 1652 cm^−1^ spectral band.

### 3.3. Classification between tissue types

The classification metrics, accuracy, sensitivity, specificity and precision, obtained after final model testing on left-out (unseen data) for MCT and STS tissues is presented in Table 1. Metrics for Raman spectral band imaging data alone are given in rows 1–3; NIR-autofluorescence imaging alone – rows 4–6; and using the combined imaging datasets – rows 7–9. For dual Raman spectral band imaging, DT achieved accuracy, precision and specificity at 86% level, followed by SVM at 83%, indicating the percentage to correctly classify between MCT or STS tumor types. For single Raman band imaging dataset, DT (or SVM) classification scores were at 78% (or at 72%) level. When using NIR-auofluorescence dataset alone, SVM achieved accuracy, sensitivity, specificity and precision above 94%. When using combined 1447 cm^−1^ and 1652cm^−1^ (1655 cm^−1^) Raman imaging and NIR-autofluorescence imaging dataset (Table 1, row 9), all classification scores achieved by SVM were close to 96%.

ROI patches with MCT or STS also provided enough discriminatory features to distinguish between cancerous and benign tissue. From Table 2 (row 3) to distinguish between MCT and benign tissue from Raman spectral band images alone, the best performing classifier was SVM with accuracy of 87.6%, sensitivity of 88.2%, specificity of 87.1%, and precision of 87.2%. To distinguish between STS and benign tissue from Raman images, the best performing classifier was also SVM with accuracy of 86.9%, sensitivity of 88.2%, specificity of 85.7% and precision of 86% (Table 3, row 3). For classification of MCT *vs* benign (or STS *vs* benign) from NIR-autofluorescence images all classification scores achieved by SVM were above 94% level (see row 6 in Tables 2 and 3).

Discussing the spectral band imaging device implementation, technically it could be applied to measure either a single spectral band (1447 cm^−1^ or 1655 cm^−1^) or sequentially acquire two spectral bands. The results of the second technical implementation are reflected in Tables 1–3, row 9. The sequential imaging of two spectral bands allows excellent (>96%) separation between the malignant and benign tissues by using the SVM classifier. However, when attempting to reduce the device complexity, price, and time interval to diagnose the tissue, the single band imaging implementation is a method of choice. For distinguishing between the tumor types, the best performing classifiers for 1447 cm^−1^ or 1655 cm^−1^ spectral band imaging are DT and SVM with 87.8–96.7% range for diagnostic metric output (Tables 1–3, rows 7, 8). Single spectral band imaging can be also used to effectively distinguish between MCT (or STS) cancer tissue and benign tissue with 87.5–92.1% (86.2–95.5%) accuracy, 85.7–92.8% (87.1–96.4%) sensitivity, 87.5–93.2% (85.3–95.7%) specificity and 87.5–93% (85.6–95.6%) precision by applying DT or SVM classifiers.

## 4. Discussion

Raman scattering offers high diagnostic accuracy to identify tumor lesions in veterinary medicine. Detection of canine malignant tissue in mammary glands and micro-calcifications (Dantas et al. 2020; Birtoiu et al. 2016; Munteanu et al. 2017) is based on peaks at 960 cm^−1^ and 1070 cm^−1^ (hydroxyapatite deposits), 1332 cm^−1^ (CH_2_/CH_3_ vibrations in collagen), 1450 cm^−1^ (CH_2_ bending mode in albumin), and 1453 cm^−1^ (structural vibration modes of proteins). For detecting malignant skin and subcutaneous tumors, in dogs and cats, Raman point spectroscopy-based methods reached sensitivity and specificity of 81.8 and 95.3% (Tamošiūnas et al. 2022). Additionally, benign tumors such as lipomas (LIP) were classified with 100% accuracy. However, the traditional single-point raster scanning approach is not ideal for a large-field-of-view Raman imaging due to its time-consuming nature when scanning areas larger than a square centimeter. The clinically feasible time range for one sample analysis is from 7 to 15 min, and the good example to fulfil the 3D sample scan in such short time interval was presented by Thomas et al. (2017). Authors investigated breast neoplasm margin status using a spatially offset Raman spectroscopy device termed ‘Marginbot’ with embedded image analysis algorithms enabling automatically reconstruct the scanned image area, and to depict the tumor margin localisation (Spigulis et al. 2021). In result, the margin scanner device achieved an accurate 3D evaluation of benign lesion margins of 3–6 cm size by applying the raster scanning. However, focusing the excitation spot can lead to high levels of light fluency (J/cm^2^), potentially causing damage or altering tissue biomolecular state. Furthermore, the resulting raster-scan image often lacks sufficient spatial resolution to effectively compare it with tissue morphology findings.

In this study we undertook a re-evaluation of the Raman spectral band imaging technique initially introduced by Papour et al. in 2015 (Papour et al. 2015; Papour et al. 2015) to promote its use in veterinary medicine. The performance of Raman macro-imaging device undergo validation for skin malformations differentiation and diagnostic accuracy estimation. The time to scan a single Raman spectral band image was 10 s; two spectral band images were required to depict a single Raman spectral band of interest; and also 3 spectroscopic measurements per sample (60 s each) were introduced into the experimental protocol. Such diagnostic approach provided bimodal imaging of two biological tissue ingredients – tissue endogenous fluorophores and tissue scatterers – specific biomolecules being related to the same malformation. Our study’s results also showed that ML classifiers, specifically SVM and DT, were able to classify STS and MCT as tumors from Raman spectral band images alone, or combined with NIR-autofluorescence images, obtaining 81–97% diagnostic accuracy.

Such imaging approach has not been used before in veterinary oncology science. Its obvious advantage – higher coherence of the dual parametric images obtained simultaneously during a single acquisition. The question still to answer is whether or not extra-weakness of signals forming the Raman spectral band images can benefit the diagnosis of cancerous tissue in the real clinical settings, during a reasonably limited time interval allocated for animal patient measurements.

Numerous studies were performed to improve human and animal tumors diagnosis, based on Raman or autofluorescence spectroscopy data separately. The principal components related to melanin Raman bands at 1342 cm^−1^ and 1613 cm^−1^, protein bands in the range of 800–1004 cm^−1^ and amide III bands in the range of 1250–1350 cm^−1^ reveal statistically significant differences between normal skin and malignant melanoma (MM) confirmed by hystopathology (Bodanese et al. 2012; Lui et al. 2012). Yorucu et al. (2016) found a relationship between Raman spectral signatures related to protein configuration or amino acid orientation and melanoma cells invading into surrounding skin cell area within 3D tissue model (Yorucu et al. 2016). Analysis of sensitivity indicate Raman spectral peaks at 1658, 1457, 1340, 1317 cm^−1^ corresponding to amide I, lipid and protein, amide III vibrational modes being capable for discriminating basal cell carcinoma (BCC) from normal tissue *ex vivo* (Larraona-Puy et al. 2009, Baek et al. 2006). Higher lipid content in BCC was revealed by Raman spectral signatures at 1004, 1092, and 1128 cm^−1^ same as lower intensity of Amide III bands related to molecular vibrations of collagen which is degraded by matrix metalloproteinases in BCC (Nijssen et al. 2002). Spectral signatures assigned to amide I band shift from 1656 to 1589 cm^−1^ and anionic phosphinyl group symmetric stretching mode shift 1085–1048 cm^−1^ could also provide useful information for BCC skin cancer diagnostics *ex vivo* (Choi et al. 2005).

Several groups applied Raman spectroscopy *ex vivo* for prognostication of malignant tissue type. Sigurdsson et al. (2004) discriminated BCC and melanoma from actinic keratosis, pigmented nevi, and normal skin tissue; Bodanese et al. (2012) and Bratchenko et al. (2017) was able to discriminate between BCC and MM with accuracy >80%. For skin cancer clinical diagnostics, Raman spectroscopy allowed discrimination of melanoma *vs* benign pigmented lesions; also melanoma *vs* seborrheic keratosis with sensitivities between 95–99% and specificities between 15–54% *in vivo* (Lui et al. 2012). Wang et al. (2012) showed that Raman spectroscopy method is also sensitive for *in vitro* squamous cell carcinoma (SCC) cell discrimination from HaCaT or melanoma cells. Using the multivariate principal component analysis, Brauchle et al. (2014) could distinguish melanoma cells from melanocytes. Furthermore, Raman spectroscopy was capable to detect the specific melanoma cells possessing BRAF mutation (which is present up to 80% in melanocytic nevi and up to 50% in primary melanomas) and NRAS mutation found in tumours (Brauchle et al. 2014). Finally, Raman spectroscopy technique was implemented on a microfluidic device to detect tumour cells present within the blood samples (Dochow et al. 2011).

**Figure 8.**
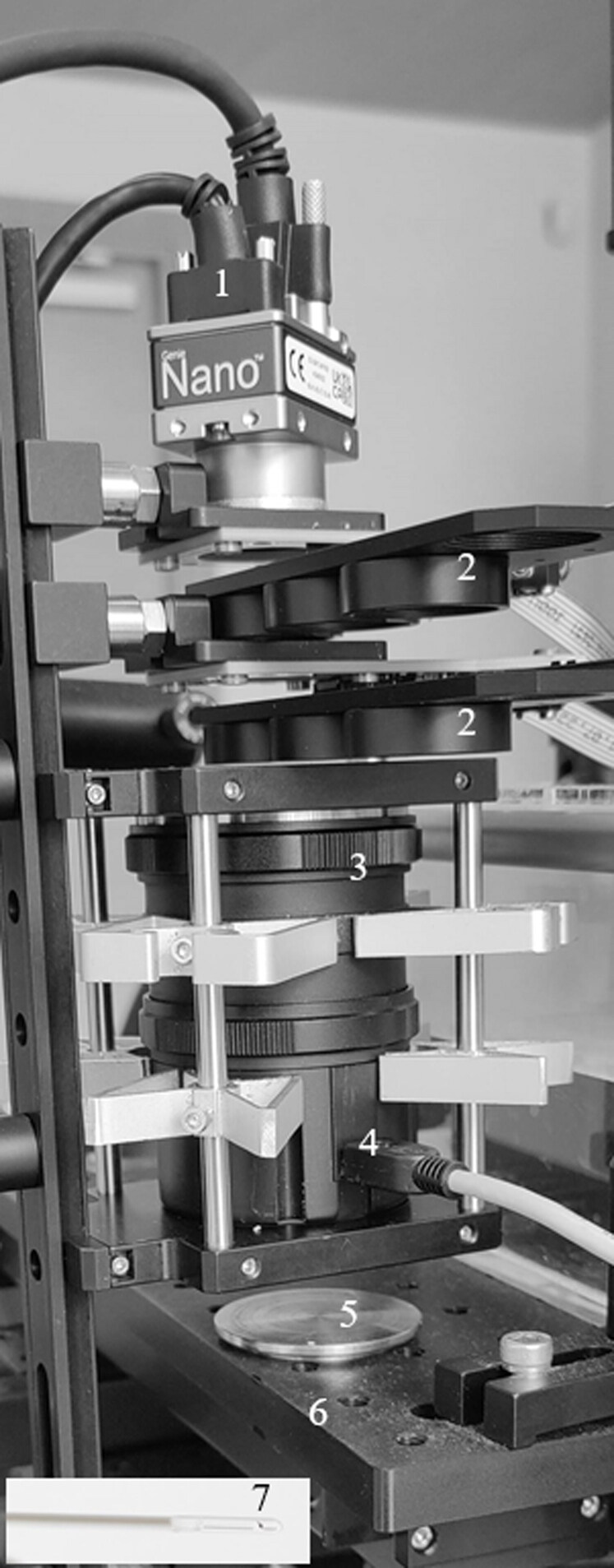
Proposed set-up for Raman + NIR-autofluorescence spectral band imaging implementation for veterinary clinics: (1) – M2590, 1” NIR camera (Teledyne Dalsa, Canada); (2) – two automated sliding filter compartments (Thorlabs, USA) equipped with calibrated rotation filers (∅1”); the proposed solution eliminated the manual rotation of two narrowband filters needed to apply during the measurement; (3) – macro imaging lens with magnification up to 5× (Yasuhara Nanoha, USA), 4 – USB powered white light LED source; 5 – sample tray (Raman signal inactive); 6 – x-y-z translation stage (Standa, Lithuania). A low-cost fiber-coupled Raman laser (FC-D-785-450 mW, CNI Optoelectronics Technology Co., Ltd, China) is used for Raman excitation *via* a 30-degree side-fire fiber probe (7), produced by Lightguide (Latvia). The probe is positioned with an additional tilting angle between the objective and tissue specimen, allowing 785 nm Raman excitation light to be delivered to the sample without interfering with the detection optics.

To increase the efficiency of cancer detection, Raman spectroscopy has been used in combination with other non-invasive optical diagnostics methods. Due to the relatively infrequent Raman scattering events, the Raman spectrum is dominated by autofluorescence signals. Autofluorescence is usually subtracted from the overall spectrum and discarded. However, this study proposes to use the autofluorescence signal as a parameter for tissue classification. Autofluorescence is the natural emission of light by biomolecules, occurring due to fluorescent materials present in tissue. Similar to Raman data, it also provides information about the tissue’s biochemical and biomolecular composition. To review the multimodal approaches in this direction, Raman and autofluorescence bimodal technologies had only been implemented as a spectroscopy setup. The study by Zakharov et al. (2015) showed that Raman spectroscopy and steady state autofluorescence spectroscopy techniques could be easily realized in a single optical device allowing more accurate diagnostics of skin cancer *ex vivo* (Zakharov et al. 2015). The accuracy of combined application of the Raman spectroscopy and autofluorescence analysis was significantly increased allowing MM diagnosis with sensitivity of 100% and specificity of 94%. The more recent work by the same group Bratchenko et al. (2017) showed that combining Raman spectral data with accompanying autofluorescence signal increased diagnostic accuracy when differentiating between melanoma, basal cell carcinoma, and normal skin *ex vivo*. In 2021, our Latvian team at Institute of Atomic Physics and spectroscopy led prof. J. Spigulis conducted clinical validation trial to detect human skin neoplasms by using the innovative system composed of three skin imaging technologies – multispectral diffuse reflectance imaging, autofluorescence lifetime imaging and EMCCD camera-based Raman spectral band imaging (Spigulis et al. 2021). We examined 38 malformations including junctional nevi, dermal nevi, combined nevi, seborrheic keratoses, hemangiomas, dermatofibroma and basal cell carcinoma, *in vivo*. Our pilot study demonstrated that Raman spectral band image of dermal nevus shows high contrast on the boundary between nevus and surrounding skin. We first tested 1437 cm^−1^ Raman spectral band signatures, which are also highest intensity bands, also in human skin. The Raman image of nevus, separated from NIR-autofluorescence, was segmented using 2-level k-means algorithm. Then, coefficient of variation CV=σ/μ was calculated from the pixels inside nevus and outside, where σ = standard deviation of pixel group analyzed and μ = pixel mean. CV is a measure of the spread of the pixel intensity that describes variability, relative to the mean, and was chosen because it can compare the spread of image regions that have different means. We proposed that the ratio between both CV_nevus_/CV_surrounding skin_ can be served as an independent diagnostic parameter for neoplasm differentiation applying Raman spectral band imaging. One should note that imaging of Raman bands by means of extremely sensitive camera with a set of narrowband filters at its input cannot be a sustainable solution for routine clinical applications. This camera was only temporarily rented, and a cost of EMCCD cameras monthly rent, which is 5% from the camera value (priced several tens of kEUR) appears too high for real clinical implementations. With further research, we aim to replace the EMCCD camera, and the laser used for Raman excitation with less expensive components (Figure 8), which implementation success would facilitate the transfer of scientific knowledge from biophotonics laboratory to applications in partner veterinary clinics. Empowering veterinary clinics with new diagnostics tools and services would increase their business performance and competitive advantage.

In conclusion, our pioneering study demonstrated the ML-algorithms powered implementation of Raman spectral band imaging method for veterinary medicine, enabling accurate diagnosis of oncological diseases *ex vivo*, such as mast cell tumors and soft tissue sarcomas. Combined Raman macro-imaging with near-infrared (NIR) autofluorescence as bimodal imaging approach resulted in 85–95% diagnostic output (accuracy, sensitivity, specificity and precision); even when classifying between the tissues from a single spectral band (1437 cm^−1^ or 1655 cm^−1^) using support vector machine and decision tree classifiers.

## Data Availability

The datasets generated and/or analyzed during the current study are available from the corresponding author upon reasonable request.

## Appendix A. Information about tumor samples used in the study.

**Table:**

#	Species	Breed	Age	Tumour	Grade of malignancy	Localization
1	Dog	French Bulldog	11 y.o.	MCT	High grade of malignancy	Skin, preputium
2	Dog	Pug	8 y.o.	MCT	Index of mitosis 2	Subcutis, chest
3	Dog	Dachshund	5 y.o.	MCT	High grade of malignancy	LNN, neck
4	Dog	Jack Russell Terrier	6 y.o.	MCT	Low grade of malignancy	Skin, scrotum
5	Dog	Mix	10 y.o.	MCT	n/a	Skin, shoulders
6	Dog	Golden Retriever	3 y.o.	MCT	Low grade of malignancy	Skin, shoulders
7	Dog	Staffordshire Bull Terrier	9 y.o.	MCT	Low grade of malignancy (x2); high grade of malignancy – big	Skin, back (x2), axillary region (big sample)
8	Dog	Jack Russell Terrier	11 y.o.	MCT	Low grade of malignancy	Skin, scapular region
9	Cat	Sphynx	n/a	MCT	n/a	Skin, neck
10	Dog	Cane Corso	5 y.o.	MCT	Index of mitosis <1	Subcutis, frontal region
11	Dog	Retriever	7 y.o.	MCT	Low grade of malignancy; 2. grade	Skin, metatarsal region
12	Dog	French Bulldog	9 y.o.	MCT	Low grade of malignancy	Skin, ear
13	Dog	American Bulldog	9 y.o.	MCT	Low grade of malignancy	Skin, hind leg
14	Dog	American Staffordshire Terrier	4 y.o.	MCT	Low grade of malignancy	Skin, ventral part of chest
15	Dog	German Shepherd	9	STS	II	Subcutis, left elbow
16	Cat	Mix	11	STS (hemangiosarcoma)	n/a	Subcutis, scapular region
17	Dog	Cane Corso	7	STS	II	Subcutis, chest
18	Dog	Mix	2	STS (hemangiosarcoma)	n/a	Skin, subcutis, knee
19	Dog	Mix	10	STS	II	Skin, elbow
20	Dog	Labrador Retriever	3	STS	III	Subcutis, elbow
21	Dog	Mix	12	STS	II	Hind leg
22	Cat	Mix	11	STS	II	Subcutis, chest
23	Cat	Mix	13 y.o.	STS (fibrosarcoma)	III	Subcutis, left hind leg
24	Dog	Mix	12 y.o.	STS	III	Subcutis, perianal region
25	Dog	Doberman Pinscher	6 y.o.	STS	I	Skin, subcutis, let forelimb
26	Cat	British Shorthair	9 y.o.	STS (fibrosarcoma)	II	Subcutis, between shoulders
27	Cat	Mix	5 y.o.	STS (fibrosarcoma)	II	Subcutis, caudal part of back
28	Cat	Domestic Shorthair	9 y.o.	STS (fibrosarcoma)	II	Subcutis, right inguinal region

## Appendix B

### 1. Workflow for separating Raman spectral band images from NIR autofluorescence

Spectral Data Analysis was conducted in Origin Lab Software.

- Raman and autofluorescence intensity assessment:
  - Measure Raman peak intensities around 1437 cm^−1^ and 1653 cm^−1^ after polynomial curve subtraction.
  - Measure autofluorescence peak intensities at 1437 cm^−1^ and 1653 cm^−1^ using the residual polynomial data curve.
- Intensity Ratios:
  - Calculate the intensity ratios between autofluorescence and Raman peak both at 1437 cm^−1^ and 1655 cm^−1^ wavenumbers.
  - Calculate the intensity ratio between the Raman spectral bands at 1437 cm^−1^ and 1655 cm^−1^.

### 2. Separation of Raman and autofluorescence spectral band images

Image Analysis (in Andor Solis Software)

1. For 1437 cm^−1^ spectral band:
  - Import ‘0-R’ and ‘R-R’ files.
  - Equalize the scale range for both image files to 0–65535 a.u.
  - Set the background of both ‘0-R’ and ‘R-R’ images to 0.
  - Test Subtraction:
    - Subtract ‘0-R’ and ‘R-R’ images using the intensity multiplicator k = 1.69 for the ‘R-R’ image. Such numeric value is derived from calibration data (Figure 2(b)), by dividing spectral intensity integral values calculated for ‘0-R’ and ‘R-R’ filer positions.
    - If artifacts appear (e.g. Raman image intensity at the laser focus point is below 0, or the ratio between autofluorescence and Raman image intensities at the focus point does not match the spectral ratio), manually adjust the ‘R-R’ image intensity multiplicator.
      - Procedure for manual adjustment:
        - Assess the average intensity values of both ‘0-R’ and ‘R-R’ images at the spot of laser excitation.
        - Adjust the ‘R-R’ image multiplicator (k) – for the spectral band image ratio to match the spectral intensity ratio.
    - Obtain the interim Raman spectral band image at 1437 cm^−1^ by subtracting modified ‘R-R’ image from ‘0-R’ image.
      - Check that the interim Raman image intensity baseline at the laser excitation spot is above 0. If not, iteratively adjust the ‘R-R’ image multiplicator (k).
      - Check that interim Raman image intensity is lower than autofluorescence but corresponds to the spectral intensity ratio.
      - If necessary, subtract a constant number to bring the autofluorescence and interim Raman image intensity background values to 0.
      - Assess the average Raman image intensity value, ensuring it corresponds to the spectral values, measured on a 16-bit intensity scale. If not, divide the interim Raman image by a constant, *C*.
    - Obtain autofluorescence image at 1437 cm^−1^:
      - Subtract the interim Raman spectral band image at 1437 cm^−1^ from the ‘0-R’ image.
      - Assess the average autofluorescence image intensity value, ensuring it corresponds to the spectral values measured on a 16-bit intensity scale. If not, divide the autofluorescence image by a constant (C) previously used for interim Raman image division.
      - If the constants for dividing Raman and autofluorescence images differ, restart the iterative subtraction process to produce interim Raman and autofluorescence spectral band images at 1437 cm^−1^ wavenumber. If the constants (C) are similar – the final Raman spectral band images and autofluorescence spectral band images are obtained.
  - Workflow differences for separating 1655 cm^−1^ Raman spectral band images from autofluorescence:
    - Use ‘R-0’ and ‘0-0’ image files, instead of ‘0-R’ and ‘R-R’, respectively.
    - Use initial intensity multiplicator k = 1.36 for ‘0-0’ image which is derived from calibration data considering 1655 cm^−1^ spectral band (Figure 2(b)).
    - Use the spectral intensity ratio values between autofluorescence and Raman peaks at 1655 cm^−1^.
    - Assess the intensity ratio between the Raman spectral bands at 1437 cm^−1^ and 1655 cm^−1^ and check for its resemblance with the spectral data.
